# In-feed oxolinic acid induces oxidative stress and histopathological alterations in Nile tilapia *Oreochromis niloticus*

**DOI:** 10.1016/j.toxrep.2025.102020

**Published:** 2025-04-04

**Authors:** Thangapalam Jawahar Abraham, Masud Bora, Avishek Bardhan, Arya Sen, Ratnapriya Das, Ranjit Kumar Nadella, Prasanna Kumar Patil

**Affiliations:** a Department of Aquatic Animal Health, Faculty of Fishery Sciences, West Bengal University of Animal and Fishery Sciences, Chakgaria, Kolkata, West Bengal 700094, India; b Fish Processing Division, ICAR-Central Institute of Fisheries Technology, Willington Island, Cochin, Kerala 682029, India; c Aquatic Animal Health and Environment Division, ICAR-Central Institute of Brackishwater Aquaculture, Raja Annamalai Puram, Chennai, Tamil Nadu 600028, India

**Keywords:** Aquaculture, Antibiotic-feed, Histopathology, Oral administration, Oxidative stress

## Abstract

The aquaculture industry urgently requires effective bacterial disease management strategies, necessitating better regulation of antibiotic application. This study investigated the effects of oral oxolinic acid (OA) administration on *Oreochromis niloticus* at the recommended dose of 12 mg (1 ×) and overdose of 36 mg (3 ×)/kg biomass/day for 7 consecutive days in terms of growth, oxidative stress, residue accretion and histopathology relative to the control. The 1 × and 3 × groups experienced dose-dependent mortalities (3.33–8.33 %). The OA residues peaked in the liver and kidney tissues with dosing and declined upon discontinuation. The residues persisted in the kidney even on day 35 post-dosing. Elevated malondialdehyde and total nitric oxide levels signified oxidative stress and correlated with the tissue level changes in various organs. Histologically, glycogen-type vacuolation and cellular hypertrophy were observed in the liver. The kidney had hydropic swelling, renal epithelium degradation, nephrocalcinosis, vacuolation, and necrosis. Splenic alterations were confined to necrosis and a slight increase in sinusoidal space. Intestinal tissues exhibited a depletion of absorptive vacuoles, epithelial layer degradation, mucinous degeneration, and necrosis. Gills displayed epithelial hyperplasia, thickening of secondary lamellae, and erosion. Nevertheless, the cohort administered the recommended dose exhibited recovery with OA discontinuation. However, none of the assessed parameters normalized in the overdosed group even after 35 days of dose suspension. The results indicated that *O. niloticus* can safely adapt to and tolerate the toxic effects of OA. As the recommended dose of OA elicited reversible bioresponses effectively in tilapia, it can be utilized in aquaculture with due caution following regulations.

## Highlights

• Oxolinic acid (OA) caused oxidative stress and histopathology in tilapia.
• A dose-dependent increase in OA residues in the liver and kidney tissues was noted.
• The recommended OA dose elicited reversible bioresponses effectively.
• Tilapia adapted to and tolerated OA overdose safely.

## Graphical Abstract

**Figure:**
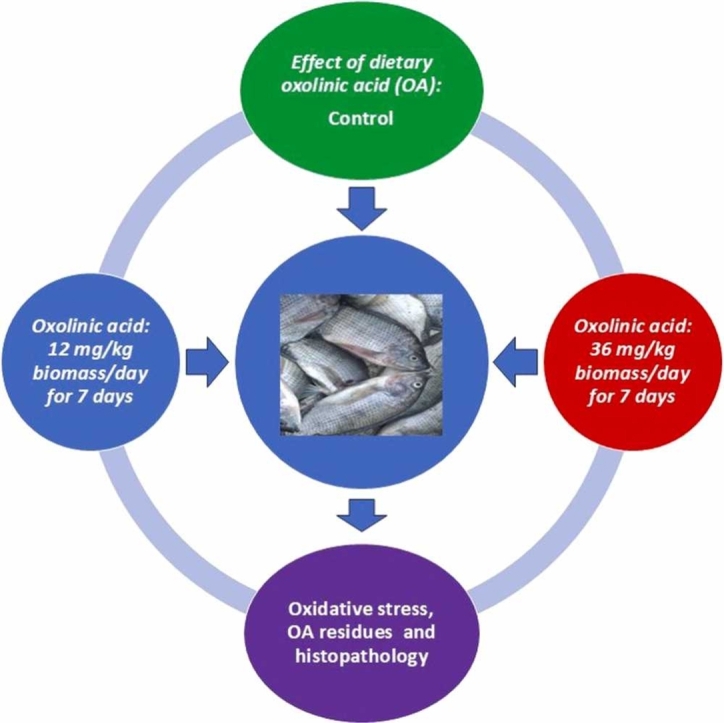

## 1 Introduction

Global aquaculture is projected to continue its robust growth, driven by increasing demand for seafood and advancements in farming technologies. Tilapia production, particularly of Nile tilapia *Oreochromis niloticus*, is expected to play a significant role, contributing approximately 8 % of global aquaculture output, with genetically improved strains enhancing growth rates and disease resistance [1]. Adopting genetically enhanced strains, such as Genetically Improved Farmed Tilapia (GIFT), has significantly improved growth rates and production efficiency, helping to meet the rising domestic and international demand for tilapia [2]. Nanotechnology and eco-friendly compounds are revolutionizing the aquaculture industry by improving disease management and feed efficiency. Nanoparticles, encapsulated vaccines, natural biopolymers, and plant-derived compounds enhance growth and health [3]. In addition, sensor technologies like wearable sensors and electronic noses monitor water quality and volatile organic compounds, ensuring optimal conditions for aquatic life. These technologies have potential in healthcare and environmental monitoring, enhancing human and animal life quality through innovative solutions [4]. With advancements in technology and a growing market for aquaculture products, the future of tilapia farming appears bright [2]. However, disease outbreaks and environmental sustainability challenges necessitate continuous research and innovation to ensure the industry's long-term success [5]. Disease management remains a significant challenge in tilapia farming, as various diseases can impact productivity and sustainability. The misuse of antibiotics in aquaculture has contributed to resistance development, complicating disease treatment and raising public health concerns due to the potential transfer of resistant bacteria through the food chain [6], [7], [8]. Pathogens such as *Aeromonas hydrophila, Streptococcus agalactiae, Edwardsiella ictaluri* and others have shown significant antibiotic resistance [9], [10], [11]. Additionally, antibiotic residues in tilapia can be a public health concern, worsening resistance, and food quality issues [12]. Addressing these challenges is critical, and adopting improved management practices, including prudent antibiotic use, and exploring alternatives will be essential for sustainable tilapia farming [13].

Oxolinic acid (OA), a first-generation fluoroquinolone antibiotic, has proven effective in managing bacterial diseases in tilapia aquaculture, particularly those caused by pathogens like *A. hydrophila* and *E. tarda* [14], [15]. Its mechanism of action involves inhibiting bacterial DNA gyrase, an enzyme critical for DNA replication, thereby limiting bacterial growth in infected fish [16]. A perusal of the literature indicated that OA is widely used in major aquaculture-producing nations [17], [18], [19], and several fish bacterial pathogens have developed resistance to potential aquaculture antibiotics [20], [21]. As per the World Organization of Animal Health, fluoroquinolones including OA should not be used as a first-line treatment unless justified, when used as a second-line treatment, it should ideally be based on the results of bacteriological tests, and extra-label/off-label use should be limited and reserved for instances where no alternatives are available [22]. However, its use is currently prohibited by Indian regulatory authorities in shrimp aquaculture [23]. There appears to be a lack of knowledge on the effects of OA on cultured fish in the Indian and Southeast Asian context. Hence, to ensure its responsible and regulated use in finfish aquaculture, further research is required on its biosafety, pharmacokinetics, and withdrawal period across various farmed aquatic species. Such studies would provide the scientific basis for developing guidelines to ensure the safe and effective use of the drug while minimising its environmental impact. This study, thus, focused on evaluating the effects of in-feed OA on the residue accretion, lipid peroxidation and histopathological alterations in the vital organs of *O. niloticus* juveniles to provide insights into its safety and potential application in aquaculture systems as a second-line treatment when primary treatment fails.

## 2 Materials and methods

### 2.1 Experimental setup and design

*Oreochromis niloticus* juveniles (21.26 ± 0.04 g; 12.75 ± 0.15 cm) were procured from Sonarpur fish farm, West Bengal, India. Upon arrival, the experimental fish underwent decontamination by immersion in 2-ppm potassium permanganate solution for 5 min before introduction into the circular tanks containing 300-L chlorine-free aerated borewell water and acclimatized for 15 days at 50 fish/tank with continuous aeration. The fish were given commercially available floating pellet feed twice daily, equivalent to 2 % body weight (BW). Any uneaten feed, faeces, or other wastes were removed daily by siphoning. About 50 % of the water exchange was done in three-day intervals. Juveniles without infections were randomly chosen from the stock tanks and placed 40 each in nine tanks. The fish were assigned into three groups, viz., Group 1 as control (0 ×); Group 2, as the recommended dose group that received 12 mg OA/kg biomass/day (1 ×) [24] and Group 3, as the overdosed group that received 36 mg OA/kg biomass/day (3 ×), in triplicates. Throughout the experimental period, the ranges of water quality parameters such as pH (7.50–7.80), dissolved oxygen (5.39–5.47 mg/L), water temperature (24.00–32.00°C), nitrite (0.17–0.20 mg/L), and nitrate (0.28–0.33 mg/L) were sustained optimally.

### 2.2 Oxolinic acid (OA) feed preparation and administration

The OA (Sigma-Aldrich, India, CAS-No: 14698–29–4) dose was determined to provide an estimated inclusion rate of 0, 12, and 36 mg/kg biomass/day. The OA feeds were prepared by feed top-dressing as described in Abraham *et al.* [25]. The required amount of OA was mixed in 5 mL soybean oil and mixed thoroughly with 1 kg basal pellet feed in an airtight container. The control feed was prepared as above but without OA. After proper mixing, the OA and control feeds were spread separately, dried overnight under the fan, and stored in airtight containers at room temperature. The fish groups were fed the respective feeds at 2 % BW thrice daily in equal rations. From day 1–7 (pre-dosing), all groups were fed with the control feed. Group 1 was fed with the control feed throughout the experiment. During the 7 days of OA-dosing (OD) period (Days 8–14), 1 × and 3 × feeds were fed to groups 2 and 3, respectively. All groups were fed the control feed during the 35 days (Days 15–49) post-OA-dosing (POD) period. The unconsumed feed, if any, in each tank after an hour of each feeding was removed, air-dried, pooled day-wise and weighed carefully. Observations on mortality, behaviour, pigmentation, and dermal lesions on the skin were recorded daily. The biomass was determined weekly to adjust the feed quantity and to assess the growth rate by estimating the biomass difference.

### 2.3 Collection of blood and plasma

Sampling was done with minimum handling stress to the experimental fish on days 0 and 7 of OD and days 14 and 35 POD. Two fish/tank were promptly anaesthetized by placing them in clove oil (40 μL/L) mixed with water [25]. The blood was drawn using 2 mL sterile syringes via a caudal puncture and transferred to 1.5 mL Eppendorf tubes rinsed with 5 % ethylenediamine tetraacetic acid (EDTA) to prevent coagulation [26]. The blood was then centrifuged at 4500 rpm for 15 min at 30°C. The resultant plasma was shifted to Eppendorf tubes and well-preserved at −20°C.

### 2.4 Assessment of lipid peroxidation and total nitric acid

The lipid peroxidation, represented by plasma malondialdehyde (MDA), was evaluated by the thiobarbituric acid reactive substances (TBARS) assay by assessing the coloured MDA-thiobarbituric acid (TBA) adduct (1:2), produced from the reaction of MDA and TBA [27]. The MDA was quantified using a microplate reader (Dynamica, Australia) at 535 nm following the kit guidelines (HiMedia, India). The nitric oxide estimation kit (HiMedia, India) was used for the total nitric oxide (TNO) assay, which relied on nitrate reduction (NO_3_) by a reducing agent at 37°C. The resultant nitrite and the endogenous nitrite were converted to a blue-coloured azo compound with Griess reagent [28]. The plasma TNO was determined spectrophotometrically at 540 nm, and computed following the kit instructions.

### 2.5 Quantification of oxolinic acid residues

The fish, after blood collection, were euthanized by increasing the clove oil dose to 100 μL/L, carefully removed the kidney and liver tissues, washed gently in flowing water to remove the blood traces and homogenized. The acidified acetonitrile (ACN) was utilized for OA extraction, involving a solvent mixture comprising 5 mL n-hexane, 10 mL 0.05 M EDTA, and 10 mL 0.1 % formic acid in ACN. The process included vortexing for a minute, incubating in a shaker at 650 rpm for 30 min, and centrifuging at 4000 rpm for 10 min at 4°C. Aliquots were purified using an Oasis hydrophilic-lipophilic balance (HLB) solid-phase extraction (SPE) cartridge for the OA analyte. The elution from the SPE method was analyzed by reverse-phase liquid chromatography on a C18 LC column. The analytical procedure relied on QTRAP LC-MS/MS. As detailed in our earlier research [25], the detection was done through electrospray ionization and tandem mass spectrometry on an ion trap mass spectrometer, and separation via a Raptor C18 2.7 µm; 100 × 2.1 mm (Restek). The in-house validation concept led to validation using diverse matrices as per the Commission Decision 2002/657/EC [19]. The method was validated at the National Referral Laboratory, ICAR-Central Institute of Fisheries Technology, Kochi, India. Validation parameters were determined based on data from mass spectrometry analysis utilizing Analyst 1.6.3 version. The response was measured in terms of peak area, exhibiting satisfactory linearity of analytes with an r^2^ value > 0.99. All the analytes showed minimal carryover (<0.5 %) in matrix-based calibration.

### 2.6 Histopathology and qualitative assessment

For histopathology, the liver, kidney, spleen, intestine, and gills were collected on OD-days 0 and 7, and POD-days 14 and 35, and fixed in Bouin’s solution for 48 h. Standardized procedures were followed for tissue processing, embedding, cutting into 5 μm sections, and performing double staining with hematoxylin and eosin, following the guidelines of Roberts [26]. Photomicrography was captured using an Olympus microscope (BX51) set with a 16 MP camera (SCO-LUX). Image acquisition and processing were carried out using ToupView software (ToupTek-x64, 4.11). The major histopathological changes in different organs were identified and qualitatively assessed on a six-point ordinal scale, according to the damage in the respective tissues from their normal architecture [29].

### 2.7 Statistical analyses

Data on MDA, TNO, and OA residues in mean ± standard deviation were analyzed by one-way ANOVA. The significance of differences among treatments and dosing periods was ascertained using the Tukey post-hoc test for mean comparisons. The non-parametric Kruskal–Wallis test assessed the qualitative scores of histopathological changes with pair-wise comparisons at p < 0.05 using IBM-Statistical Package for Social Sciences, Version 22.0.

## 3 Results

### 3.1 Feed intake, abnormalities, mortalities, and biomass

The control group exhibited vigorous and assertive feeding behaviour. During the OD tenure, feed intake was reduced by 1–3 % in the 1 × and 3 × groups. With dose termination, it steadily increased and almost reached 100 % in both groups. The fish displayed no aberrant behaviour, except for the increased production of skin and gill mucus in the 3 × group. During the OD phase, mortalities recorded in the 1 × and 3 × groups were 3.33 ± 1.44 and 8.33 ± 1.44 %, respectively, which differed significantly. On day 35 POD, insignificantly higher mortalities were noted in both groups. There were significant variations in the biomass among groups, with the 3 × group showing the lowest growth rate.

### 3.2 Lipid peroxidation, total nitric acid production and oxolinic acid residues

The plasma MDA showed a dose-dependent significant increase with the highest on day 7 of OD (p < 0.05). The recommended dose group accomplished normalcy on day 14 POD, while the 3 × group returned to normal on day 35 POD. The plasma TNO increased significantly and peaked on day 7 (p < 0.05), with the 3 × group displaying the highest level. With dose suspension and time, the TNO reduced significantly (p < 0.05). The 1 × group was normalized, and the 3 × group failed to recover on day 35 POD (Table 1). The OA residues in the liver and kidney had a significant accumulation that reached its highest on day 7 of OD. The OA residues depleted with dose suspension and were below the detectable level on day 14 of POD in the 1 × group’s liver. In the kidney, the residues remained detectable even on day 35 POD in both groups (Table 1).

**Table 1:** Plasma thiobarbituric acid reactive substances (TBARS), total nitric oxide (TNO), and liver and kidney tissue oxolinic acid (OA) residues in OA-dosed *Oreochromis niloticus*.

Plasma oxidative stress markers				OA residues				
Treatment day	TBARS (µM)		TNO (µM)		Liver (µg/kg)		Kidney (µg/kg)	
	1 ×	3 ×	1 ×	3 ×	1 ×	3 ×	1 ×	3 ×
Day 0	1.16 ± 0.05	1.16 ± 0.05	61.15 ± 0.58	61.15 ± 0.58	0.00 ± 0.00	0.00 ± 0.00	0.00 ± 0.00	0.00 ± 0.00
Day 7 OD	1.41 ± 0.05*	1.54 ± 0.05^*@^	138.75 ± 1.00*	172.50 ± 1.15^*@^	35.60 ± 1.00*	94.20 ± 0.10^*@^	94.23 ± 0.95*	270.30 ± 0.61^*@^
Day 14 POD	1.22 ± 0.05	1.27 ± 0.05*	97.50 ± 1.00*	132.50 ± 1.10^*@^	BDL	1.33 ± 0.01	2.17 ± 0.07	4.37 ± 0.02
Day 35 POD	1.16 ± 0.05	1.17 ± 0.05	62.50 ± 0.58	82.50 ± 0.72^*@^	BDL	BDL	1.76 ± 0.02	2.60 ± 0.10

OD: Oxolinic acid dosing; POD: Post-oxolinic acid-dosing; TBARS: Thiobarbituric acid reactive substances as malondialdehyde (MDA); TNO: Total nitric oxide; BDL: Below detectable limit; 1 × : 12 mg/kg biomass/day; 3 × : 36 mg/kg biomass/day. *: Significant increase compared to day 0 (p < 0.05); @: Significant increase compared to the 1 × group (p < 0.05).

### 3.3 Histopathology

**Fig. 1:**
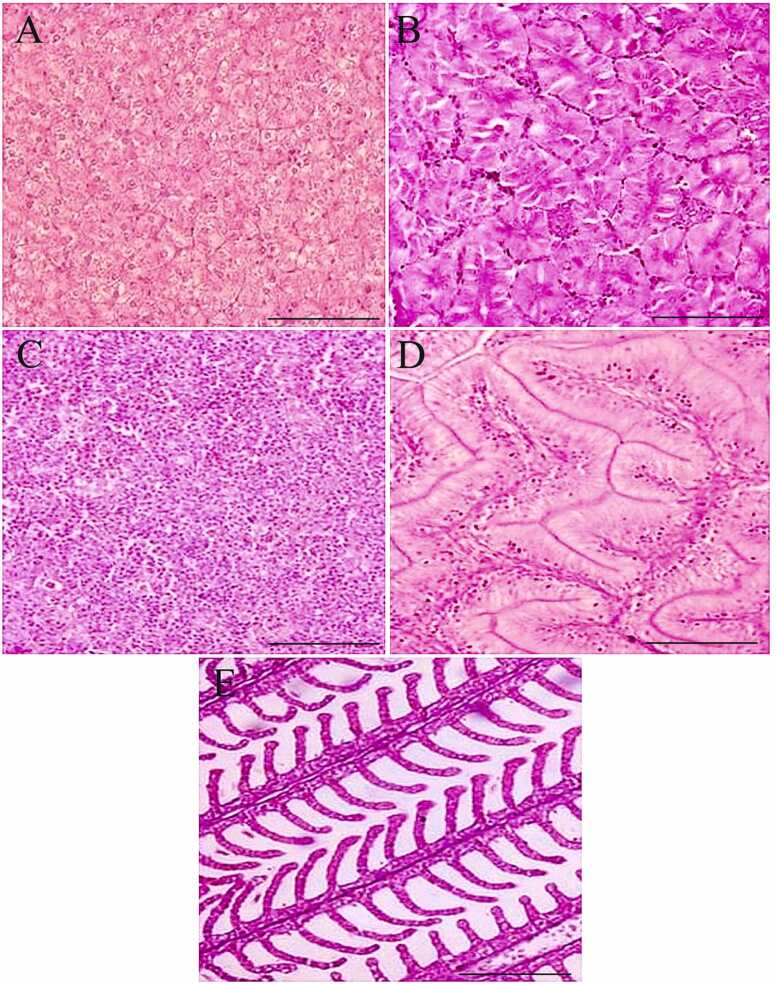
Normal histoarchitecture of the control feed-fed *Oreochromis niloticus* showing [A] liver, [B] kidney, [C] spleen, [D] intestine and [G] gill. × 200H&E staining; Scale Bar: 100 µm.

**Fig. 2:**
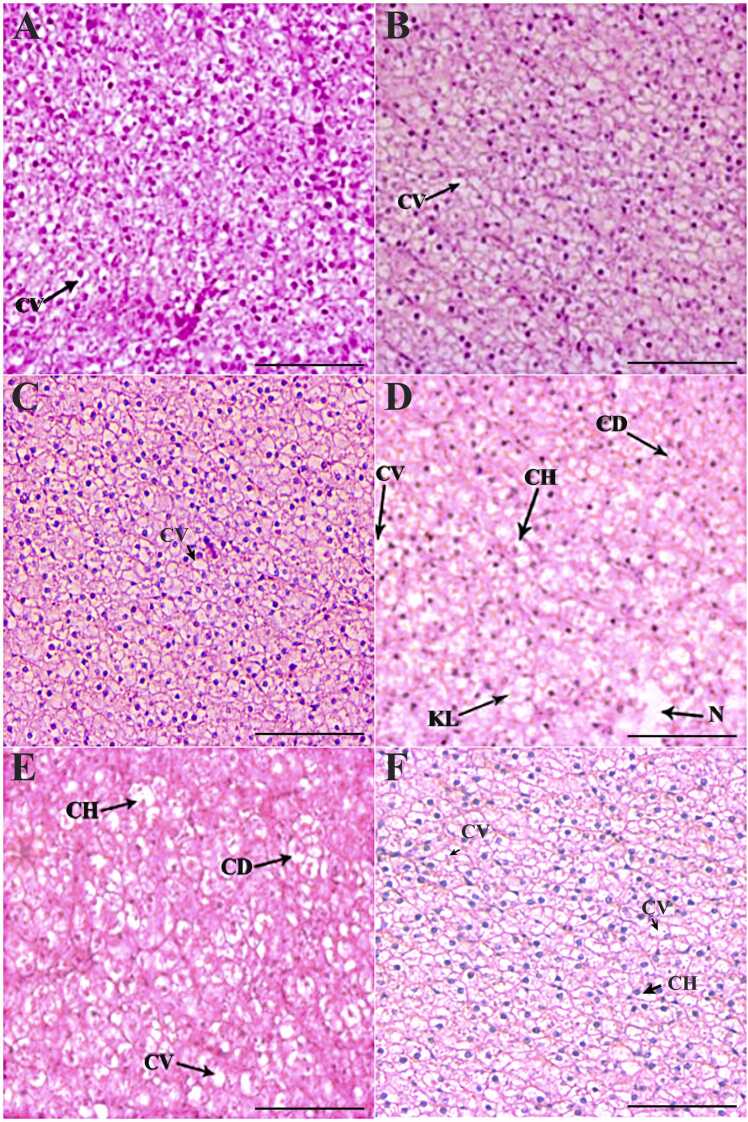
Histopathological changes in the liver of oxolinic acid (OA)-fed *Oreochromis niloticus* at 12 mg (1 ×) and 36 mg/kg biomass/day (3 ×) for 7 consecutive days. [A] 1 × group on day 7 of OD, [B] day 14 POD, [C] day 35 POD, [D] 3 × group on day 7 of OD, [E] day 14 POD, and [F] day 35 POD. OD: OA-dosing; POD: Post-OA-dosing. Cytoplasmic vacuolation (CV), cytoplasmic degeneration (CD), cellular hypertrophy (CH), karyolytic nuclear abnormalities (KL) and necrosis of liver tissue (N). × 200H&E staining; Scale Bar: 100 µm.

**Fig. 3:**
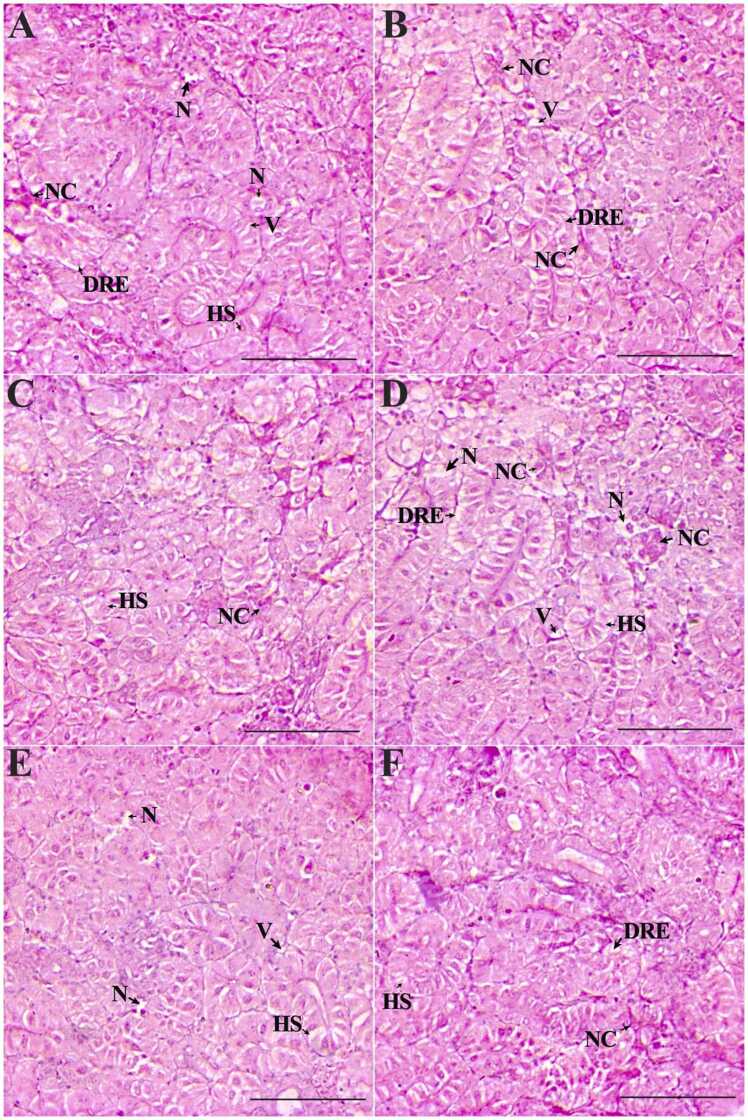
Histopathological changes in the kidney of oxolinic acid (OA)-fed *Oreochromis niloticus* at 12 mg (1 ×) and 36 mg/kg biomass/day (3 ×) for 7 consecutive days. [A] 1 × group on day 7 of OD, [B] day 14 POD, [C] day 35 POD, [D] 3 × group on day 7 of OD, [E] day 14 POD, and [F] day 35 POD. OD: OA-dosing; POD: Post-OA-dosing. Degeneration of renal epithelium (DRE), hydropic swelling (HS), necrotized areas (N), nephrocalcinosis (NC) and vacuolation (V). × 200H&E staining; Scale Bar: 100 µm.

The normal histoarchitecture of the liver, kidney, spleen, intestine and gill tissues is presented in Fig. 1A-E. The liver tissues and hepatocytes underwent considerable changes compared to the control (Fig. 2A-F). On day 7, the 1 × group revealed significant glycogen-type vacuolation. The POD period recorded a decrease in the degree of glycogen-type vacuolation, and the liver tissue organization improved significantly (Fig. 2B-C). However, the 3 × group liver tissues displayed an increased intensity of changes, including cytoplasmic degeneration, glycogen-type vacuolation, and cellular hypertrophy, along with minor alterations like necrosis and karyolytic nuclear abnormalities (Fig. 2D). Though the liver tissues exhibited signs of recovery from day 14 POD (Fig. 2E), glycogen-type vacuolation and cellular hypertrophy were prominent on day 35 POD (Fig. 2F). Compared to the control, the OA-fed *O. niloticus* had considerable renal tissue damage (Fig. 3A-F). The 1 × group exhibited necrotic areas, vacuolation, renal epithelium impairment, hydropic swelling, and nephrocalcinosis on day 7 of OD (Fig. 3A). On day 14 POD, the renal tissues started showing recovery and the abnormalities decreased, although mild degenerative renal epithelium, nephrocalcinosis and vacuolation persisted (Fig. 3B). On day 35 POD, the intensity of nephrocalcinosis and hydropic swelling reduced (Fig. 3C). The 3 × group had a significantly higher degree of damage on day 7 (Fig. 3D). From day 14 POD, the kidney tissues showed signs of recovery and with reduced damages (Fig. 3E, F).

**Fig. 4:**
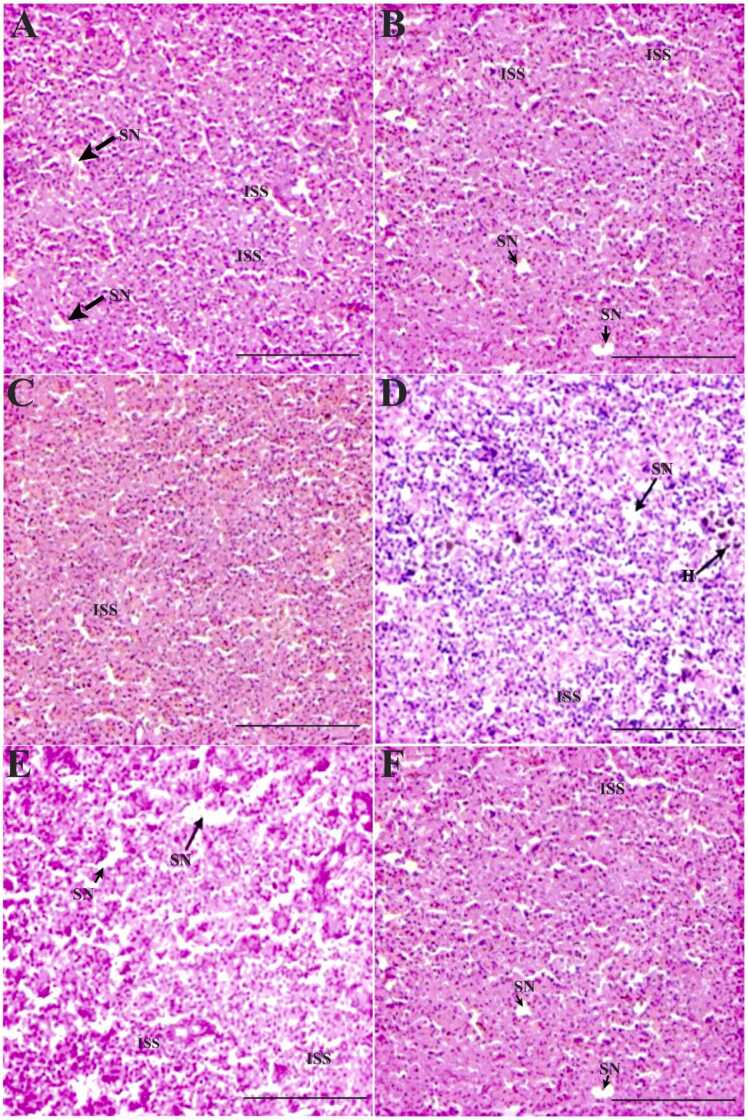
Histopathological changes in the spleen of oxolinic acid (OA)-fed *Oreochromis niloticus* at 12 mg (1 ×) and 36 mg/kg biomass/day (3 ×) for 7 consecutive days. [A] 1 × group on day 7 of OD, [B] day 14 POD, [C] day 35 POD, [D] 3 × group on day 7 of OD, [E] day 14 POD, and [F] day 35 POD. OD: OA-dosing; POD: Post-OA-dosing. Splenic necrosis (SN), increased sinusoidal space (ISS) and hemosiderin deposits (H), × 200H&E staining; Scale Bar: 100 µm.

**Fig. 5:**
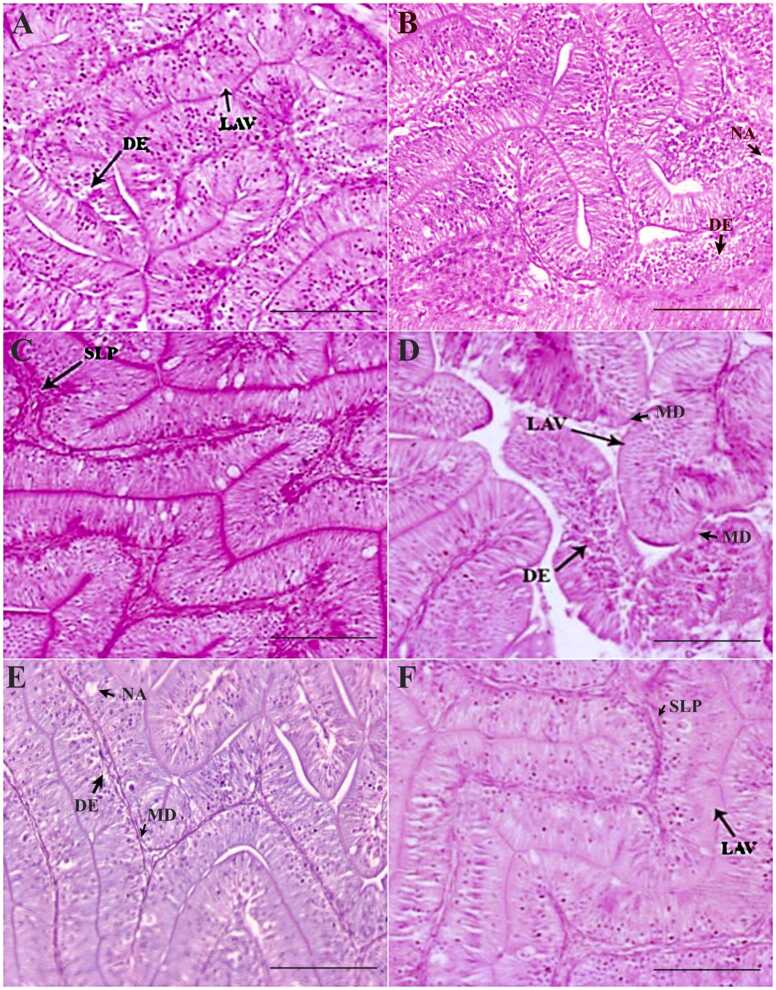
Histopathological changes in the intestine of oxolinic acid (OA)-fed *Oreochromis niloticus* at 12 mg (1 ×) and 36 mg/kg biomass/day (3 ×) for 7 consecutive days. [A] 1 × group on day 7 of OD, [B] day 14 POD, [C] day 35 POD, [D] 3 × group on day 7 of OD, [E] day 14 POD, and [F] day 35 POD. OD: OA-dosing; POD: Post-OA-dosing. Moderate loss of absorptive vacuoles (LAV), mildly degenerated epithelial layer (DE), mildly degenerated epithelial layer (DE), necrotized area (NA), mildly swollen lamina propria (SLP), and mucinous degeneration (MD), × 200H&E staining; Scale Bar: 100 µm.

**Fig. 6:**
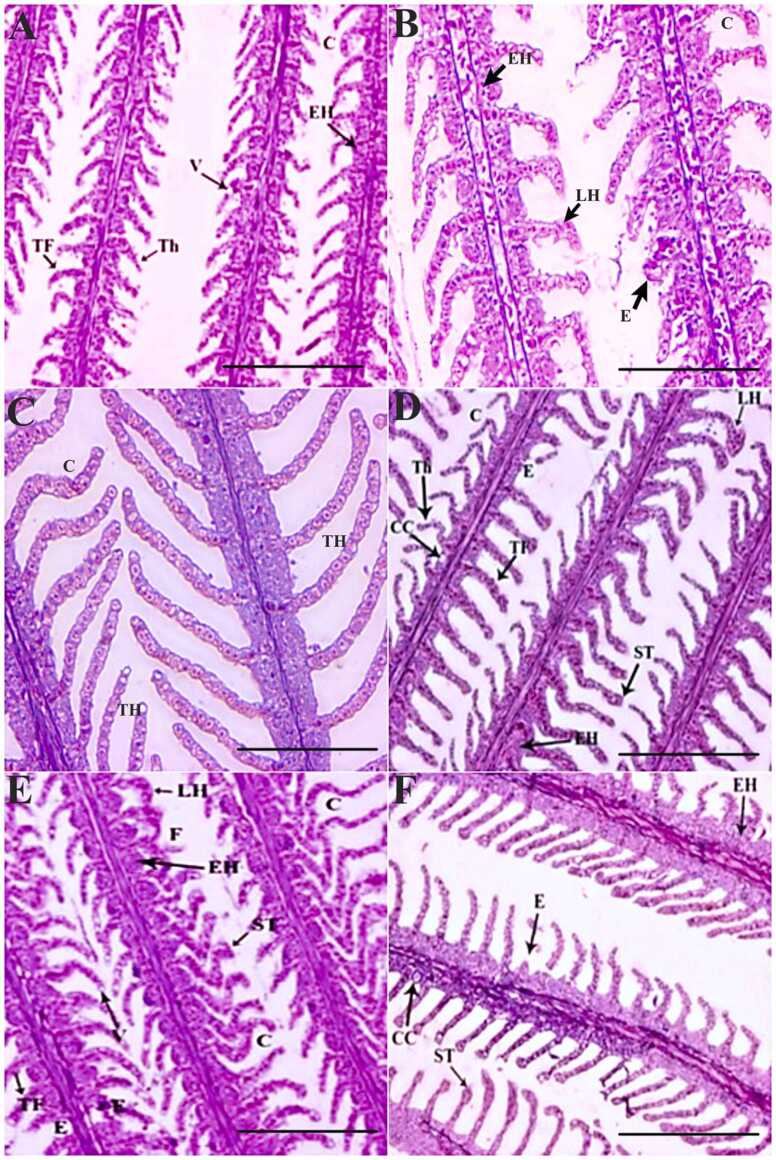
Histopathological changes in the gill of oxolinic acid (OA)-fed *Oreochromis niloticus* at 12 mg (1 ×) and 36 mg/kg biomass/day (3 ×) for 7 consecutive days. [A] 1 × group on day 7 of OD, [B] day 14 POD, [C] day 35 POD, [D] 3 × group on day 7 of OD, [E] day 14 POD, and [F] day 35 POD. OD: OA-dosing; POD: Post-OA-dosing. Epithelial hyperplasia (EH), thinning of secondary lamellae (Th), thickening of secondary lamellae (TF), curling of secondary lamellae (C), vacuolation (V), erosion of secondary lamellae (E), lamellar hyperplasia (LH), chloride cells (CC), swollen tips (ST), fusion of secondary lamellar filaments (F), and mild lamellar hyperplasia (LH), × 200H&E staining; Scale Bar: 100 µm.

The spleen tissues of OA-dosed *O. niloticus* demonstrated changes like increased sinusoidal space, splenic necrosis, and hemosiderin deposits in a dose-dependent fashion. The 1 × group’s spleen on day 7 demonstrated splenic necrosis and mildly increased sinusoidal space (Fig. 4A) In the 3 × group, these alterations were significantly (p < 0.05) severe (Fig. 4D). On days 14 and 35 of POD, both the groups’ spleen tissue showed significant (p < 0.05) recovery despite the persistence of increased sinusoidal space and splenic necrosis (Fig. 4B, C), although the recovery was less in 3 × group compared to the 1 × group (Fig. 4E, F). Oral OD for 7 consecutive days caused considerable alterations in the intestine of *O. niloticus*. The 1 × group documented minor changes like loss of absorptive vacuoles and degenerated epithelial layer on day 7 of OD compared to the control (Fig. 5A). On day 14 of POD, the 1 × group showed trivial recovery (Fig. 5B). However, on day 35 POD, recovery of tissue histoarchitecture was considerable in the 1 × group with significant (p < 0.05) reduction in the aberrations to an almost normal level, except for the swollen lamina propria (Fig. 5C). In the 3 × group, significant (p < 0.05) alterations such as loss of absorptive vacuoles, mucinous degeneration and degenerated epithelial layer were observed on day 7 (Fig. 5D). On day 14 POD, significant (p < 0.05) recovery was noted, which continued till day 35 POD, although the swollen lamina propria and mucinous degeneration were persistent (Fig. 5E, F). The gills of the 1 × group recorded epithelial hyperplasia, thinning of secondary lamellae, curling of secondary lamellae, and thickening of secondary lamellae on day 7 of OD compared to the control (Fig. 6A). On day 14 POD, a significant reduction (p < 0.05) and recovery in the gill tissues were observed (Fig. 6B). On day 35 POD, significant reductions (p < 0.05) and recovery in the gill tissues was observed with the persistent minor alterations like curling and thickening of secondary lamellae (Fig. 6C). The 3 × group showed a significant hike in alterations compared to the 1 × group (Fig. 6D). On day 14 and 35 POD, significant reduction in alterations (p < 0.05) and recovery of the gill tissues were observed with substantial persistence of swollen tips of the secondary lamella, erosion of secondary lamellae, epithelial hyperplasia, and chloride cells (Fig. 6E, F). The qualitative scores of the major histopathological changes in the vital organs of OA-dosed *O. niloticus* juveniles are presented in Table 2.

**Table 2:** Qualitative assessment of the major histopathological changes in oxolinic acid (OA)-dosed *Oreochromis niloticus* juveniles at 12 mg (1 ×) and 36 mg/kg biomass/day (3 ×) for 7 consecutive days in comparison with normal architecture.

Histopathological changes	Qualitative assessment on a six-point ordinal scale*					
	1 ×			3 ×		
	7 OD	14 POD	35 POD	7 OD	14 POD	35 POD
Liver						
Glycogen-type vacuolation	2.70 ± 0.45^a^	2.25 ± 0.45	1.85 ± 0.42	3.30 ± 0.22^@^	2.75 ± 0.36	2.45 ± 0.15
Cytoplasmic degeneration	1.25 ± 0.25^a^	0.85 ± 0.20	0.55 ± 0.15	1.85 ± 0.25^@^	1.30 ± 0.35	0.85 ± 0.20
Cellular hypertrophy	1.85 ± 0.25	1.85 ± 0.25	1.85 ± 0.25	1.50 ± 0.26^@^	1.20 ± 0.20	0.85 ± 0.15
Kidney						
Degeneration of renal epithelium	1.75 ± 0.15^a^	0.40 ± 06	0.25 ± 06	2.45 ± 0.20^@^	0.65 ± 0.21	0.45 ± 0.06
Hydropic swelling	1.35 ± 0.30^a^	0.55 ± 0.20	0.35 ± 0.08	1.55 ± 0.26^@^	0.85 ± 0.15	0.65 ± 0.15
Nephrocalcinosis	1.00 ± 0.05^a^	0.65 ± 0.25	0.50 ± 0.22	1.25 ± 0.16^@^	0.70 ± 0.18	0.50 ± 0.18
Vacuolation	0.45 ± 0.15^a^	0.35 ± 0.12	0.25 ± 0.12	1.30 ± 0.10^@^	0.75 ± 0.22	0.65 ± 0.21
Spleen						
Splenic necrosis	0.85 ± 0.10^a^	0.65 ± 0.10	0.45 ± 0.11	1.15 ± 0.10^@^	0.85 ± 0.10	0.65 ± 0.11
Increased sinusoidal space	1.15 ± 0.12^a^	0.75 ± 0.12	0.55 ± 0.16	1.65 ± 0.08^@^	1.05 ± 0.12	0.75 ± 0.16
Intestine						
Loss of absorptive vacuoles	1.75 ± 0.15^a^	0.30 ± 0.11	0.20 ± 0.10	2.10 ± 0.05^@^	0.47 ± 0.10	0.35 ± 0.10
Degenerated epithelial layer	0.75 ± 0.12^a^	0.65 ± 0.08	0.45 ± 0.08	1.00 ± 0.06^@^	0.85 ± 0.11	0.65 ± 0.08
Necrotized area	0.30 ± 0.15^a^	0.30 ± 0.15	0.25 ± 0.08	0.85 ± 0.20^@^	0.85 ± 0.18	0.45 ± 0.10
Swollen lamina propria	1.35 ± 0.08	1.35 ± 0.08	1.35 ± 0.08	2.10 ± 0.05^@^	1.75 ± 0.15	1.65 ± 0.15
Mucinous degeneration	0.65 ± 0.10^a^	0.45 ± 0.08	0.25 ± 0.05	0.85 ± 0.15^@^	0.60 ± 0.10	0.40 ± 0.08
Gill						
Thinning of secondary lamellae	0.55 ± 0.24^a^	0.35 ± 0.15	0.25 ± 0.15	0.85 ± 0.24^@^	0.65 ± 0.15	0.35 ± 0.10
Epithelial hyperplasia	1.65 ± 0.20^a^	1.05 ± 0.20	0.75 ± 0.15	1.85 ± 0.25^@^	1.25 ± 0.20	0.85 ± 0.15
Curling of secondary lamellae	0.35 ± 0.08^a^	0.35 ± 0.08	0.25 ± 0.10	0.67 ± 0.05^@^	0.45 ± 0.10	0.25 ± 0.08
Thickening of secondary lamellae	0.53 ± 0.24^a^	0.35 ± 0.20	0.25 ± 0.15	0.53 ± 0.24^@^	0.53 ± 0.24	0.35 ± 0.20
Lamellar hyperplasia	0.45 ± 0.20^a^	0.27 ± 0.10	0.20 ± 0.10	0.60 ± 0.20^@^	0.35 ± 0.10	0.25 ± 0.15
Erosion of secondary lamellae	0.45 ± 0.20^a^	0.27 ± 0.10	0.15 ± 0.05	0.65 ± 0.10^@^	0.35 ± 0.20	0.27 ± 0.10
Swollen tips of secondary lamellae	0.25 ± 0.20^a^	0.20 ± 0.10	0.20 ± 0.08	0.45 ± 0.20^@^	0.35 ± 0.20	0.27 ± 0.10

* Qualitative assessment ordinal scale: 0 = No change; 1 = Normal with < 5 % of tissues affected; 2 = Mild with 5–15 % of tissues affected; 3 = Moderate with 15–25 % of tissues affected; 4 = Marked with 25–50 % of tissues affected and 5 = Severe with > 50 % of tissues affected. The qualitative assessment was based on six observations (mean ± standard deviation) for each organ of the respective group. No changes were noted in the control. The qualitative scores on histopathological changes between the 1 × and 3 × groups on days 7, 14, and 35 differed significantly (p < 0.05). 7 OD: Day 7 OA-dosing; 14 POD: Day 14 post- OA-dosing; 35 POD: Day 35 post-OA-dosing. a, and @: Significant increase compared to the post- OA-dosing regimen of the 1 × and 3 × groups, respectively (p < 0.05).

## 4 Discussion

*Oreochromis niloticus* administered OA orally showed a significant and dose-dependent reduction in feed intake, survival, and growth. The fish were able to mount biological responses post-dosing, and feed intake recovered. Mortalities were found in both dosing groups, indicating OA-induced stress and intoxication. These observations corroborated the works of Abraham *et al.* [25], who asserted dose and time-dependent OA intoxication in *O. niloticus* and supported the observations of Hentschel *et al.* [30] on drug dose and toxicity. The study found significant changes in fish biomass during and after OD, indicating a negative effect of OA on fish growth. The control group showed a 1.25-fold increase in biomass, while the OD groups showed a dose-dependent decrease correlated with the decrease in feed intake. The results on the elevated plasma MDA and TNO indicated that dietary OA was rapidly absorbed and disrupted the balance between oxidants and antioxidants in *O. niloticus*, causing the liver and kidney to produce excessive free radicals and metabolic and tissue damage. The heightened MDA and TNO in OA-fed *O. niloticus* suggested a decrease in the activity of antioxidant enzymes, leading to diminished antioxidant capacity corroborating the findings of Bardhan *et al*. [31] noted in florfenicol (FFC)-administered *O. niloticus*. The outcome of this study contradicted several prior studies that observed a reduction in MDA following oxytetracycline (OTC) administration [32], [33]. With dose cessation, the MDA recouped on days 14 and 35 POD in the 1 × and 3 × groups, respectively, indicating the adaptive responses of fish. As nitric oxide (NO) is a reactive nitrogen species critical in the redox biology of hepatocytes, it plays a significant role in inducing oxidative stress in fish [31]. The elevated TNO might believably be one of the primary factors contributing to the inflamed liver, particularly in the overdosed group. Following the cessation of OD, *O. niloticus* exhibited a recovery, suggesting their ability to mount adaptive responses against OA-induced stress and to initiate bioresponses to overcome the abnormalities.

During dosing, a dose-dependent build-up of OA residues was observed in the liver and kidney tissues. The presence of residues in fish caused stress, physiological disorders, and organal changes as was noted in the kidney and liver histopathology and plasma MDA and TNO levels. Depletion occurred more rapidly in the liver and kidney tissues, with the liver becoming residue-free on day 14 POD in the recommended dose group. In the overdosed group, residues were noticeable in the kidney samples on day 35 POD, thereby showing adverse safety concerns regarding the abuse of antibiotics. Chen *et al*. [17] noted that most quinolones are primarily eliminated through renal excretion and liver metabolic processes, involving both glomerular filtration and tubular secretion. This aligns with the findings of faster elimination of residues from liver and kidney tissues at the recommended dose. Overall, the *O. niloticus* in the current study displayed comparably low accrual and high removal efficiency of OA residues. Depletion results of OA in *O. niloticus* are consistent with the depletion of OA in several other species [17], [34], [35], [36]. The depletion pattern described in this work provided evidence in favour of more sensible OA use in *O. niloticus.*

The OA-dosed *O. niloticus* liver tissues showed structural irregularities, more or less, similar to the alterations noted in several fish species treated with chloroquine [37], norfloxacin [38], OA [25], [39], ciprofloxacin [40], and fluoroquinolone [41]. Their findings suggested that quinolone exposure/administration could lead to diverse consequences of aberrant metabolic processes, potentially leading to liver failure. The glycogen-type vacuolation was the most significant and prominent histopathological aberration, indicating an increased demand for energy to overcome the OA-induced stress. It further supported the histological observations with OTC [42], FFC [31], and OA [25]. Vacuolations suggested diverse biochemical changes, including hindered protein synthesis and ionic regulation, decreased energy levels, enzyme denaturation, alterations in substrate utilisation, and disruption of microtubules in the liver [37]. The severe damage to the liver was due to the hyperactivity of the nucleus, which can be indicated by cellular hypertrophy, nuclear degeneration, alteration of hepatocytes, and necrosis. As the liver is the primary source of exoenzymes such as alanine aminotransferase (ALT), aspartate aminotransferase (AST), and alkaline phosphatase (ALP) bound to the membranes of hepatocytes, the damage to the liver tissue may release these membrane-bound proteins from the impaired hepatocytes. In several earlier studies, fish subjected to antibiotics such as OTC, FFC, OA and others had higher ALT, AST, and ALP levels, which indicated their hepatotoxic effect [25], [31], [42], [43]. On day 35 POD, the liver tissues displayed insufficient improvement, characterized by the persistence of vacuolation with glycogen-type features, despite the below detectable levels of residues. Nonetheless, the recommended dose group improved tissue histoarchitecture suggesting their ability to initiate physiological responses to overcome these abnormalities. The recovery of cellular hypertrophy was only close to normal even on 35 days of POD, thus suggesting the persistence of OA-induced stress in the liver.

The present study recorded significant and dose-dependent histopathological changes in the kidney on day 7. Nephrotoxic effects of OA were recognized by the degeneration of renal epithelium, hydropic swelling and vacuolation, which corroborated previous studies on fish treated with ciprofloxacin [40], chloroquine [37], OA [25], FFC [31] and OTC [44]. Degeneration is recognized as a non-specific condition that acts as an initial indication of necrosis [45]. Furthermore, the heightened presence of disintegrated renal epithelium and vacuolation indicated that vacuolation precedes disintegration [45]. On day 7, scanty nephrocalcinosis was observed, which may be associated with a decrease in calcium and impaired reabsorption [25]. As the kidney is involved in the calcium and phosphate homeostasis processes, its disturbances may lead to mineral deposition within the renal tubules [46]. Inflammation of the renal tubules can occur in higher vertebrates, and it can be acute or chronic, depending on the primary cell type or cell response involved [45]. The hydropic swelling of epithelial cells could potentially be the source of inflammation of the renal tubules. Although subtle, the increase in hydropic swelling was dose-dependent. These changes proclaimed a dose-dependent nephrotoxic effect of OA in *O. niloticus.* However, the changes were reversible as observed on day 35 POD. The fish administered OA displayed noticeable changes in the spleen tissue. A mild to moderate increase in sinusoidal space and splenic necrosis, indicating the toxic effects of OA on the fish spleen. It possibly affected the spleen's functioning as the sinusoids reportedly remove damaged and old erythrocytes as well as transfer leucocytes [47]. Splenic necrosis occurs when the blood supply to the spleen is reduced, resulting in tissue ischemia and, finally, necrosis [40]. In *Oncorhynchus mykiss* congested cells, foci of myocyte necrosis, and small clusters of melanocytes were noticed upon enrofloxacin treatment [48]. Hemosiderin is an iron-storing pigment that is derived from the breakdown of erythrocytes [49]. In the current study, hemosiderosis was found due to the toxicity of OA, indicating the reduction of erythrocytes. Reduced erythrocytes and hemosiderosis have a link with splenic necrosis [49]. The recommended dose group experienced mild splenic alterations that returned to normal after the medication was terminated, demonstrating the tolerance of OA at the recommended dose. Further, the study suggested that OA has mild to moderate toxic effects on the spleen of *O. niloticus* and may cause reversible histopathological alterations.

The histological alterations and impairments identified in intestinal tissues of *O. niloticus* due to OD in varying degrees were dose-dependent and attributed to the direct toxicity of OA. Epithelial layer degeneration was observed in all OD groups, ranging from normal to mild, throughout the dosing and post-dosing periods, possibly due to the drug rejection by the fish intestinal lining. A mild increase in degeneration of epithelium proclaimed that the harmful potential of OA on the intestinal tissues was the least. The mild to moderate increase in the loss of absorptive vacuoles seen throughout the OD duration indicated poor feed absorption and, as a result, reduced feed intake. The loss of absorptive vacuoles was most observed in all OD groups throughout the experimental period, revealing the reduction in energy charges as the intestine expends a lot of energy during digestion and absorption [50], [51]. The loss of such energy charge in the intestinal villi may be responsible for epithelial cell destruction. The lamina propria is a vascularized connective tissue that exists beneath the epithelium and is composed of nerves and leukocytes [52]. An increase in lamina propria thickness indicated heightened immunity and the host responses to drug absorption [52]. The dose-dependent alterations, like mucinous degeneration and necrotized area in the intestinal villi upon OD, were only normal to mild. Kitamura *et al.* [53] stated that quinolones can reduce mucus-producing goblet cells, and promote intestinal disruption and inflammation. The main function of intestinal goblet cells is the production of mucin and the formation of mucus layers for innate defence [54], and their reductions may result in intestinal damage and breach of innate defences. In the intestine of *O. mykiss*, enrofloxacin treatment caused an increased number of eosinophilic granular leukocytes [48]. Except for the persistence of mucinous degeneration and swollen lamina propria, all other aberrations recovered to normal, demonstrating tolerance of *O. niloticus* to OA at the recommended dose.

Modifications in the gill morphology can potentially suffocate fish by disrupting normal respiratory function and preventing an adequate supply of oxygen and inorganic ions from achieving the fish's normal metabolic processes [40]. Xu *et al.* [55] reported that quinolones interfere with fish respiratory and osmoregulation activities, and essential gill functions to induce gill inflammation and oxidative stress. This study documented several structural anomalies in the recommended dose group on day 7 with increased intensity in the overdosed group, similar to fish treated with chloroquine [37] and ciprofloxacin [41]. Epithelial and secondary lamellar hyperplasia may develop from some defence cells, such as macrophages and leucocytes, as part of a compensatory mechanism of tissue healing [56]. The curling and swollen tips of secondary lamellae were noticed during the dosing period, leading to improper oxygen uptake and anoxia. The swelling of secondary lamellar filaments may cause the reduction of oxygen consumption efficiency of fish gills [31], [57]. On day 35 POD, except for epithelial hyperplasia, other anomalies returned to normal or imperfectly recovered, with mild alterations. A comparison of the results of the present study with previous publications on quinolone compounds on varied fish species is presented in the supplementary Table. This study, thus, demonstrated that OA can cause mild to moderate structural and functional damage in the vital organs of fish, which is a cause for concern.

## 5 Conclusion

The present study demonstrated that oral administration of OA induces dose-dependent physiological and histopathological alterations, significantly affecting *O. niloticus* health. The reduction in feed intake, growth, and survival highlighted the adverse effects of OA overdose. The study further confirmed that OA administration induces oxidative stress, as evidenced by elevated MDA and TNO levels, leading to metabolic and tissue damage in the liver and kidney. While the fish exhibited an ability to recover post-dosing, the histopathological analysis of the liver, kidney, spleen, intestine, and gills revealed persistent structural impairments, particularly in the overdosed group. The OA residues peaked on day 7 of OD and declined with dose suspension. Residue diminution occurred more rapidly, with the liver becoming residue-free on day 14 POD in the recommended dose group. The residues were detectable in both groups’ kidneys on day 35 POD. The accumulation of OA residues in the liver and kidney tissues further indicated the potential health risks associated with OA use in aquaculture. The observed depletion patterns suggested that responsible and controlled use of OA is necessary to mitigate long-term health implications. The increasing concern regarding antimicrobial resistance emphasized the importance of using OA cautiously as second-line therapy in food-producing aquatic animals when there are no other options available, as it could potentially pose risks to fish health. Overall, the present study contributed significantly by providing a detailed assessment of OA’s dose-dependent impact on fish health, highlighting recovery patterns post-administration, and offering histopathological evidence of tissue damage in the vital organs. The findings on OA residue depletion provide valuable insights for food safety regulations, reinforcing the need for responsible antibiotic use and the adoption of sustainable aquaculture practices. Nevertheless, additional research on the long-term effects of OA administration on fish, the environmental impact of OA residues, the combined toxic effect of OA with other chemicals or water quality parameters in the aquaculture systems, the precise molecular mechanisms driving OA-induced oxidative stress, and the potentials of vaccines, probiotics or herbal alternatives to improve the disease resistance may yield some directives on the effective mitigation strategies for bacterial disease control. Therefore, further research should explore sustainable fish health management approaches, ensuring aquaculture productivity without compromising fish welfare and consumer safety.

## CRediT authorship contribution statement

**Nadella Ranjit Kumar:** Validation, Resources, Investigation. **Das Ratnapriya:** Investigation, Formal analysis, Data curation. **Sen Arya:** Writing – original draft, Investigation, Formal analysis, Data curation. **Bardhan Avishek:** Investigation, Formal analysis, Data curation. **Bora Masud:** Investigation, Formal analysis, Data curation. **Thangapalam Jawahar Abraham:** Writing – review & editing, Supervision, Resources, Project administration, Methodology, Conceptualization. **Patil Prasanna Kumar:** Supervision, Methodology, Funding acquisition, Conceptualization.

## Ethics approval

The current study was performed following the guidelines of the Committee for the Purpose of Control and Supervision of Experiments on Animals (CPCSEA), Government of India. The experimental protocols were approved by the ICAR, Government of India, New Delhi, under the All-India Network Project on Fish Health (F. No. CIBA/AINP-FH/2015–16 dated 16.7.2015).

## Funding

10.13039/501100001503Indian Council of Agricultural Research (ICAR), Government of India, New Delhi, under the All-India Network Project on Fish Health, supported this work vide Grant F. No. CIBA/AINP-FH/2015–16 dated 02.06.2015.

## Declaration of Competing Interest

The authors declare that they have no known competing financial interests or personal relationships that could have appeared to influence the work reported in this paper.

## Data Availability

Data will be made available on request.

## Footnotes

Appendix A Supplementary data associated with this article can be found in the online version at doi:10.1016/j.toxrep.2025.102020.
