# Supplementary material for: In-feed oxolinic acid induces oxidative stress and histopathological alterations in Nile tilapia Oreochromis niloticus
Source: Toxicol Rep. 2025 Apr 4;14:102020. doi: 10.1016/j.toxrep.2025.102020 (PMC12002751; doi:10.1016/j.toxrep.2025.102020)
Supplement: Supplementary file 1 — Supplementary material [file mmc1.docx]

**Supplementary Table 1.** A comparison of the results of the present study with previous publications on quinolone compounds on varied fish species

| Organ | Present study (*Oreochromis niloticus*) | Fish species | Drug | Changes observed | References |
| --- | --- | --- | --- | --- | --- |
| Liver | Glycogen-type vacuolation, cytoplasmic degeneration, and cellular hypertrophy | *Cyprinus carpio* | Norfloxacin | Adipose tissue near the liver sinusoids, and nuclear enlargement | [38] |
|  |  | *Oncorhynchus mykiss* | Oxolinic acid | Nuclear enlargement, cytoplasmic disorganisation, increase in rough endoplasmic reticulum, and lipid deposition | [39] |
|  |  | *Cirrhinus mrigala* | Ciprofloxacin | Cellular oedema, fatty infiltration, nuclei degeneration, and vacuoles | [40] |
|  |  | *Cyprinus carpio* | Chloroquine* | Nuclear degeneration, vacuolization, cellular oedema, increased sinusoidal space, necrosis, and altered hepatocyte | [37] |
|  |  | *Oreochromis niloticus* | Oxolinic acid | Cytoplasmic vacuolation, cellular hypertrophy, cytoplasmic degeneration, karyolytic nuclear abnormalities, and necrosis | [25] |
|  |  | *Danio rerio* | Fluoroquinolone | Charyopyknosis, cholestasis, and lipid droplet deposition | [41] |
| Kidney | Degeneration of renal epithelium, hydropic swelling, nephrocalcinosis, and vacuolation | *Cirrhinus mrigala* | Ciprofloxacin | Degeneration and hypertrophy, fragmented glomerulus, tubular necrosis, and vacuolation | [40] |
|  |  | *Cyprinus carpio* | Chloroquine* | Thickening of Bowman’s capsule, tubular cell necrosis, shrinkage of glomeruli, tubular degeneration, glomerular necrosis, hyaline droplet degeneration, cloudy swelling, congestion in renal parenchyma, reduction of lumens, renal tubular separation, and necrosis | [37] |
|  |  | *Oreochromis niloticus* | Oxolinic acid | Necrosis, inflammation, hydropic swelling, nephrocalcinosis, vacuolation, and degeneration of the renal epithelium | [25] |
| Spleen | Splenic necrosis and increased sinusoidal space | *Oncorhynchus mykiss* | Enrofloxacin | Congested cells, mild per-acute to acute foci of myocyte necrosis, and small clusters of melanocytes | [48] |
| Intestine | Loss of absorptive vacuoles, degenerated epithelial layer, necrotized area, swollen lamina propria, and mucinous degeneration | *Oncorhynchus mykiss* | Enrofloxacin | Increased number of eosinophilic granular leukocytes | [48] |
|  |  | *Oreochromis niloticus* | Oxolinic acid | Degeneration of intestinal epithelium, loss of absorptive vacuoles, mucinous degeneration, necrotized intestinal areas, and swollen lamina propria | [25] |
| Gill | Thinning of secondary lamellae, epithelial hyperplasia, curling of secondary lamellae, thickening of secondary lamellae, lamellar hyperplasia, erosion of secondary lamellae, and swollen tips of secondary lamellae | *Cirrhinus mrigala* | Ciprofloxacin | Cytoplasmic vacuolation, epithelial lifting, lamellar fusion, loosening of primary gill bar, and necrosis | [40] |
|  |  | *Cyprinus carpio* | Chloroquine* | Lamellar fusion, degenerative and necrotic changes in the epithelium of gill filaments, oedema, vacuolization, hyperplasia, blood congestion, epithelial lifting, hypertrophy, fusion, and curling | [37] |

*: Chloroquine is a member of the drug class 4-aminoquinoline used as an antimalarial drug.
